# The Eagle effect in the *Wolbachia*-worm symbiosis

**DOI:** 10.1186/s13071-020-04545-w

**Published:** 2021-02-24

**Authors:** Christina A. Bulman, Laura Chappell, Emma Gunderson, Ian Vogel, Brenda Beerntsen, Barton E. Slatko, William Sullivan, Judy A. Sakanari

**Affiliations:** 1 Department of Pharmaceutical Chemistry, University of California, San Francisco, CA USA; 2 Department of Molecular, Cell and Developmental Biology, University of California, Santa Cruz, CA USA; 3 Veterinary Pathobiology, University of Missouri-Columbia, Columbia, MO USA; 4 Molecular Parasitology Division, New England Biolabs Inc, Ipswich, MA USA

**Keywords:** *Wolbachia*, Eagle effect, Endosymbiosis, Filaria

## Abstract

**Background:**

Onchocerciasis (river blindness) and lymphatic filariasis (elephantiasis) are two human neglected tropical diseases that cause major disabilities. Mass administration of drugs targeting the microfilarial stage has reduced transmission and eliminated these diseases in several countries but a macrofilaricidal drug that kills or sterilizes the adult worms is critically needed to eradicate the diseases. The causative agents of onchocerciasis and lymphatic filariasis are filarial worms that harbor the endosymbiotic bacterium *Wolbachia.* Because filarial worms depend on *Wolbachia* for reproduction and survival, drugs targeting *Wolbachia* hold great promise as a means to eliminate these diseases.

**Methods:**

To better understand the relationship between *Wolbachia *and its worm host, adult *Brugia pahangi* were exposed to varying concentrations of doxycycline, minocycline, tetracycline and rifampicin *in vitro* and assessed for *Wolbachia *numbers and worm motility. Worm motility was monitored using the Worminator system, and *Wolbachia* titers were assessed by qPCR of the single copy gene *wsp* from *Wolbachia *and *gst *from *Brugia *to calculate IC_50_s and in time course experiments*.* Confocal microscopy was also used to quantify *Wolbachia *located at the distal tip region of worm ovaries to assess the effects of antibiotic treatment in this region of the worm where *Wolbachia *are transmitted vertically to the microfilarial stage.

**Results:**

Worms treated with higher concentrations of antibiotics had higher *Wolbachia *titers, i.e. as antibiotic concentrations increased there was a corresponding increase in *Wolbachia *titers. As the concentration of antibiotic increased, worms stopped moving and never recovered despite maintaining *Wolbachia *titers comparable to controls. Thus, worms were rendered moribund by the higher concentrations of antibiotics but *Wolbachia* persisted suggesting that these antibiotics may act directly on the worms at high concentration. Surprisingly, in contrast to these results, antibiotics given at low concentrations reduced *Wolbachia *titers.

**Conclusion:**

*Wolbachia *in *B. pahangi *display a counterintuitive dose response known as the “Eagle effect.” This effect in *Wolbachia *suggests a common underlying mechanism that allows diverse bacterial and fungal species to persist despite exposure to high concentrations of antimicrobial compounds. To our knowledge this is the first report of this phenomenon occurring in an intracellular endosymbiont, *Wolbachia*, in its filarial host.
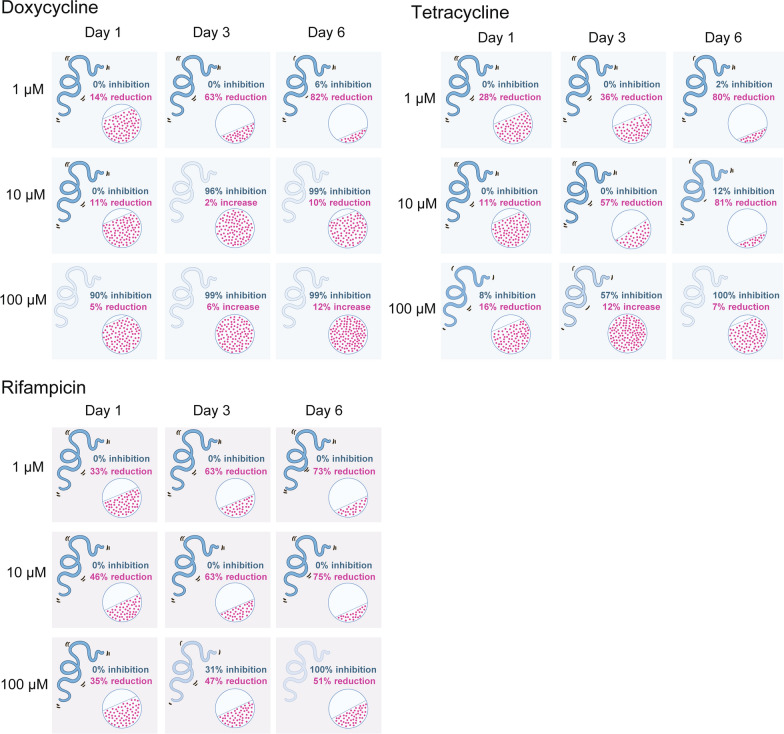

## Background

Onchocerciasis and lymphatic filariasis are two human neglected tropical diseases caused by parasitic filarial nematodes. Onchocerciasis, also known as river blindness, is caused by *Onchocerca* *volvulus*, while lymphatic filariasis is caused by the species *Wuchereria bancrofti*, *Brugia malayi* and *Brugia timori*. Each of these species harbors the endosymbiotic bacterium, *Wolbachia,* in the hypodermal chord and female ovaries, where the endosymbiont is passed through the female germline [1]. These filarial worms depend on *Wolbachia* for their long-term survival and reproduction, and *Wolbachia* also play a role in the clinical pathology of filarial infection [2–9]. The microfilaricidal drug ivermectin, which has been successfully used in mass drug administration (MDA) programs to eliminate onchocerciasis in Central and South America [10, 11], cannot be used in Central and West Africa because of the severe adverse effects in patients co-infected with high numbers of *Loa loa* microfilariae [12, 13]. *Loa loa*, unlike *Onchocerca*, *Wuchereria* and *Brugia*, does not harbor *Wolbachia* [14, 15], thus identifying antibiotics that eliminate *Wolbachia* is an excellent approach to find new drugs to eliminate onchocerciasis and lymphatic filariasis [16–19].

Clinical studies have shown that doxycycline given to patients for 4–6 weeks at 100–200 mg/day was efficacious in reducing disease pathology and microfilaremia in individuals with lymphatic filariasis [20–22] and was also effective in reducing *Wolbachia*, disrupting worm fertility and causing adult worm death in patients infected with *O. volvulus* [23–26]. Although effective as an anti-*Wolbachia* drug, doxycycline is contraindicated during pregnancy and for young children, and the long course of treatment is not feasible for MDA because of the challenges of patient adherence [15, 27–31]. Antibiotics such as rifampicin and minocycline, as well as novel anti-*Wolbachia* drugs, have also shown promise in pre-clinical models of lymphatic filariasis and onchocerciasis [19, 32–37]. However, there is evidence in pre-clinical models that if insufficient anti-*Wolbachia* treatment is administered, *Wolbachia* can repopulate their host leading to recovery of filarial fecundity [35, 38, 39].

Much remains unknown about the mechanisms by which *Wolbachia* repopulates an antibiotic-treated filarial worm and how the filarial worm regains its reproductive output. While it is clear that *Wolbachia* and its filarial host are co-dependent, the mechanisms by which *Wolbachia* abundance influences worm viability is unknown. This information is critical for both understanding the biology of the *Wolbachia*-worm symbiosis and developing efficacious protocols for treating these devastating diseases. Because of the high costs and difficulties associated with animal studies, *in vitro* studies have provided an excellent means to study the *Wolbachia/Brugia* relationship. Here we tested several antibiotics, doxycycline, tetracycline, minocycline, rifampicin and two novel anti-wolbachial compounds, with adult *B. pahangi* females and males *in vitro* to determine *Wolbachia* titers and their effects on worm viability. Surprisingly, there was a positive correlation between antibiotic concentrations and *Wolbachia* titers, a phenomenon known as the “Eagle effect,” where higher concentrations of antibiotics correlate with increased titers of bacteria [40–43]. We also determined that antibiotics affected worm viability without first reducing *Wolbachia* titers, suggesting that these antibiotics may act directly on the worms *in vitro* at high concentration.

## Methods

### *Brugia pahangi* worm assays and motility assessment

Adult *B. pahangi* female and male worms were collected from jirds (*Meriones unguiculatus*) and transferred to 24-well plates with 500 µl of culture media (RPMI-1640 with 25 mM HEPES, 2.0 g/L NaHCO_3_, 5% heat-inactivated FBS and 1X antibiotic/antimycotic solution). To limit variability among individual female worms, only fecund female worms that released at least 50 microfilariae (mf) were used. To determine IC_50_s, worms were treated with a 6-point serial dilution of 100, 30, 10, 3, 1 and 0.3 µM of doxycycline hyclate (Sigma-Aldrich catalog no. D9891), minocycline hydrochloride (Sigma-Aldrich catalog no. M9511), tetracycline hydrochloride (Sigma-Aldrich catalog no. T7660) or rifampicin (Fisher Scientific catalog no. 50-213-645). To avoid precipitation of the antibiotics in media, we used a maximum concentration of 100 μM, which is below the limit of solubility in water for each of the antibiotics [44–47]. One percent DMSO (Fisher Scientific catalog no. BP231) was used for the control worms. Female worms were plated individually, and male worms were plated four per well. Worms were kept in culture in a 37 °C, 5% CO_2_ incubator for the duration of the assay (6 days). Worm motility was recorded on Days 0, 1, 2, 3 and 6 using the Worminator [48], and worms were collected on Day 6 for qPCR analysis.

To confirm that worm motility correlated with worm viability, *B. pahangi* females that had been treated with 100, 10 and 1 µM doxycycline were collected on Day 6 and assayed using a cell viability assay with thiazolyl blue tetrazolium bromide (MTT) (Sigma Aldrich catalog no. M2128) similar to ones used previously [49–51]. Worms were transferred to a 96-well plate containing 200 µl freshly prepared 0.5 mg/ml MTT in PBS per well, incubated at 37°C for 30 min and then transferred to 150 µl DMSO. After 1 h, 100 µl DMSO was transferred to a clear, flat-bottom 96-well plate, and the absorbance of formazan was read at 570 nm.

To compare the effects of two different classes of antibiotics on adult *Brugia*, doxycycline and tetracycline (tetracycline class of antibiotics) and rifampicin (macrocyclic antibiotic) were used in a time course experiment with male and female worms. Worms were treated with different concentrations of antibiotic and assessed over multiple time points. Female worms were treated with 100, 10 and 1 µM antibiotic, and male worms were treated with 100 and 1 µM antibiotic. DMSO (1%) was used as the negative control. Motility was recorded on Days 0, 1, 2, 3, 5 and 6, and worms were collected for qPCR analysis on Days 1, 3 and 6.

Two novel quinazoline compounds, CBR417 and CBR490 (provided by Calibr-Scripps Research Institute, San Diego, CA) [34], were tested with *B. pahangi* females at 100, 10 and 1 µM. Motility was recorded on Days 0–3, and worms were collected on Day 3 for qPCR analysis. All compounds were completely soluble at all concentrations.

### Quantification of *wsp* and *gst* copy numbers from *B. pahangi* worms

Treated worms were washed in PBS, frozen in a dry ice/ethanol bath and stored at −80 °C. Genomic DNA from individual female worms was extracted using the Qiagen DNeasy Blood & Tissue Kit, and genomic DNA from four male worms was extracted using the QIAampDNA micro kit. The *Wolbachia* surface protein (*wsp*) and *Brugia pahangi* glutathione S-transferase (*gst*) primers [52] were used with the GeneCopoeia All-in-One SYBR Green qPCR mix and run in a BioRad CRX Connect thermocycler. pCR4-TOPO plasmid standards containing *wsp* and *gst* genes were used to calculate gene copy numbers from Ct values. The following primer sequences were used: *gst*_fwd 5ʹ-GAGACACCTTGCTCGCAAAC-3ʹ; *gst*_rev 5ʹ-ATCACGGACGCCTTCACAG-3ʹ; *wsp*_fwd 5ʹ-CCCTGCAAAGGCACAAGTTATTG-3ʹ; *wsp*_rev 5ʹ-CGAGCTCCAGCAAAGAGTTTAATTT-3ʹ.

For amplification of *gst*, the reaction mix was heated at 95^o^ C for 15 min, followed by 36 cycles of denaturation at 94 °C for 15 s, annealing at 55 °C for 30 s and elongation at 72 °C for 30 s. After the final cycle, melting curve analysis was conducted by heating the reaction mix at 95^o^ C for 1 min, annealing at 55 °C for 30 s and then heating to 97 °C. For amplification of *wsp*, the reaction mix was heated to 95 °C for 15 min, followed by 40 cycles of denaturation at 94 °C for 10 s, annealing at 57 °C for 20 s and elongation at 72 °C for 15 s. After the final cycle, melting curve analysis was conducted by heating the reaction mix at 95 °C for 1 min, annealing at 55 °C for 30 s and then heating to 95 °C.

### Quantification of *Wolbachia* in distal tip region of *B. pahangi* ovaries by immunofluorescence assay

To visually confirm the effects of antibiotics on *Wolbachia*, worms were stained with immunofluorescent dyes and examined by confocal microscopy. As with previous studies [34, 53], quantification was limited to the distal tip region of the ovaries, which has a more consistent distribution of *Wolbachia* in developing oocytes than the hypodermal chords, where *Wolbachia* are often dispersed as regional accumulations of bacteria [1, 53, 54]. Female worms treated with 10 µM doxycycline, minocycline, tetracycline and rifampicin were frozen in drug-free culture media at −80 °C on Day 6 for immunofluorescence staining. Worms were thawed and immediately fixed in 3.2% paraformaldehyde for 25 min and then rinsed with PBST (PBS with 0.1% Triton-X100). Ovaries were dissected from the worm bodies and stained with propidium iodide (1 mg/ml diluted 100X in PBST) for 30 s, then mounted with DAPI VECTASHIELD mounting medium (Vector Labs) and imaged using an SP5 confocal microscope. *Wolbachia* titers were obtained by counting the number of puncta per µm^2^ area.

### Statistical analyses

Motility data were normalized to the mean motility of DMSO control worms. Motility data (percent inhibitions) were constrained to 0 and 100% inhibition [55], and IC_50_s were calculated using GraphPad Prism software (Version 8.1.2). The statistical significance of reductions in motility in the time course experiment was determined using a two-way ANOVA followed by Tukey’s multiple comparisons test.

Correlation coefficients (*r*) were determined using the CORREL function in Microsoft Excel for Mac 2011 (version 14.7.7). Correlation coefficients were determined for worm motility *vs* formazan production in the MTT assay, antibiotic concentration *vs* *wsp/gst* ratios of treated worms and worm motility *vs* *wsp/gst* ratios of treated worms.

Statistical significance of puncta per µm^2^ in the distal tip region of worm ovaries was determined using the Kruskal-Wallis test followed by Dunn’s multiple comparisons test with GraphPad Prism version 8.1.2.

To compare *Wolbachia* titers at different antibiotic concentrations in the time course experiment, *wsp/gst* ratios of treated worms were normalized to their respective DMSO controls. Statistical significance was determined using a two-way ANOVA followed by Tukey’s multiple comparison test, with comparisons across antibiotic treatments within each time point and across time points within each antibiotic treatment. Percent differences in *Wolbachia* titers compared to DMSO controls were calculated based on the medians of the treatment groups and DMSO controls.

## Results

### Worm motility is highly correlated to viability in MTT assay

To confirm that worm motility is indicative of worm viability, worms were analyzed using an MTT assay similar to ones used previously [49–51]. Results showed that cell viability as measured by the conversion of MTT to formazan was highly correlated with worm motility (*r* = 0.889) and that earlier cessation of worm motility was predictive of greater reduction in formazan production on Day 6 (Table 1) similar to the results found with *B. malayi* [49] and *O. gutturosa* [50].

**Table 1:** Viability of female worms treated with doxycycline was highly correlated with worm motility

	% Inhibition of motility					% Inhibition of formazan production
Compound	Day 1 (%)	Day 2 (%)	Day 3 (%)	Day 4 (%)	Day 6 (%)	Day 6 (%)
Doxycycline (100 µM)	54	94	97	99	99	91
Doxycycline (10 µM)	0	0	9	59	92	46
Doxycycline (1 µM)	0	0	0	2	0	8

Worms treated with 100 and 10 µM doxycycline showed declining motility over time and were barely motile at 100 µM by Day 2. Viability was assessed on Day 6, as measured by formazan production in an MTT assay. The degree and duration of motility inhibition was predictive of reduced viability in the MTT assay

### Eagle effect in an endosymbiotic bacterium from a filarial worm

**Fig. 1:**
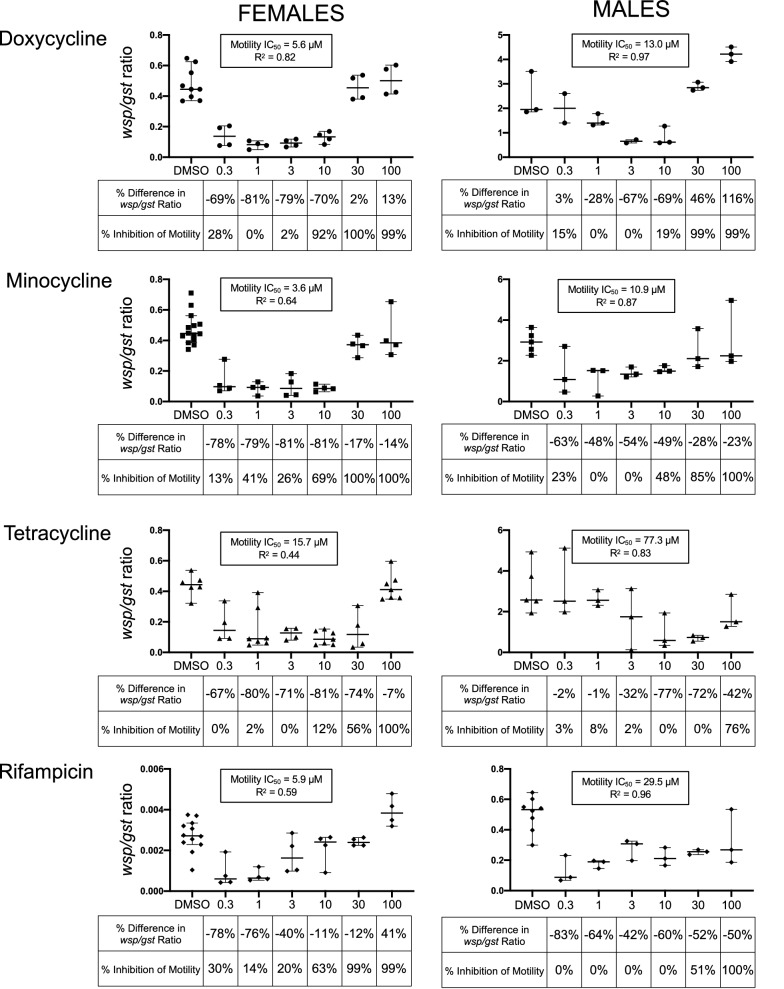
*Brugia pahangi* worms exposed to higher concentrations of antibiotics maintained higher *Wolbachia* titers. Female and male worms were treated with 6-point serial dilutions of doxycycline, minocycline, tetracycline and rifampicin *in vitro* for 6 days and assessed for worm motility and *Wolbachia* titers (see also Fig. S1 and Table S1 for statistical significance). For female worms, higher concentrations of antibiotics inhibited worm motility, but surprisingly *Wolbachia* titers did not decrease. Male worms were similarly affected except for those treated with tetracycline, which did not show a positive correlation between *Wolbachia* titers and antibiotic concentration. *Wolbachia* titers were measured by qPCR as a ratio of *wsp/gst* (shown as medians with 95% confidence intervals); antibiotic concentrations are in µM. The percent differences in *wsp/gst* ratios as compared to DMSO controls are shown below each antibiotic concentration. Negative percentages signify a decrease in *Wolbachia* titers, and positive percentages indicate titers that were higher than controls. Percent inhibition of worm motility is also shown below each antibiotic concentration: 0% inhibition indicates that worms were as motile as controls, and 100% inhibition indicates that the worms were not motile. There was an inverse relationship between worm motility and *wsp/gst* ratio (*r* ≤ −0.5), except for males treated with tetracycline, which did not show this inverse relationship

To better understand the relationship between *Wolbachia* and its worm host, adult *Brugia pahangi* were exposed to varying concentrations of doxycycline, minocycline, tetracycline and rifampicin and assessed for *Wolbachia* numbers and worm motility. Results showed that *Wolbachia* titers were significantly reduced at antibiotic concentrations that are at or slightly below the IC_50_s for worm motility in female worms (Fig. 1; Additional file 1: Fig. S1, Table S1). In contrast to these results, worms treated with higher concentrations of antibiotics had higher *Wolbachia* titers, i.e. as antibiotic concentrations increased there was a corresponding increase in *Wolbachia* titers (the Eagle effect). However, as the concentration of antibiotic increased, worms stopped moving and never recovered despite maintaining *Wolbachia* titers comparable to controls. Thus, worms were rendered moribund by the higher concentrations of antibiotics but *Wolbachia* persisted. The same trends in *Wolbachia* titers were observed when *wsp* copy numbers were analyzed both with and without normalization to worm *gst* copy numbers (Additional file 1: Fig. S2 and S3), indicating that changes in the *wsp/gst* ratio reflect changes in *Wolbachia* titer and were not driven by changes in *gst* copy number. *Wolbachia* titers in males treated with doxycycline, minocycline and rifampicin followed a similar pattern as observed in females with a positive correlation between *Wolbachia* titers and compound concentration (correlation coefficient of *r* ≥ 0.5). Male worms treated with tetracycline, however, did not show a positive correlation between *Wolbachia* titers and compound concentration (Fig. 1; Additional file 1: Fig. S1 and S3, Table S1).

The motility-based IC_50_s for doxycycline, minocycline, tetracycline and rifampicin with female worms after 6 days *in vitro* were: 5.6, 3.6, 15.7 and 5.9 µM, respectively; for male worms, the IC_50_s for each of the antibiotics were 13.0, 10.9, 77.3 and 29.5 µM, respectively (Additional file 1: Fig. S1, Table S1).

### Doxycycline and tetracycline inhibited worm motility without reducing *Wolbachia* titers in time course experiments

**Fig. 2:**
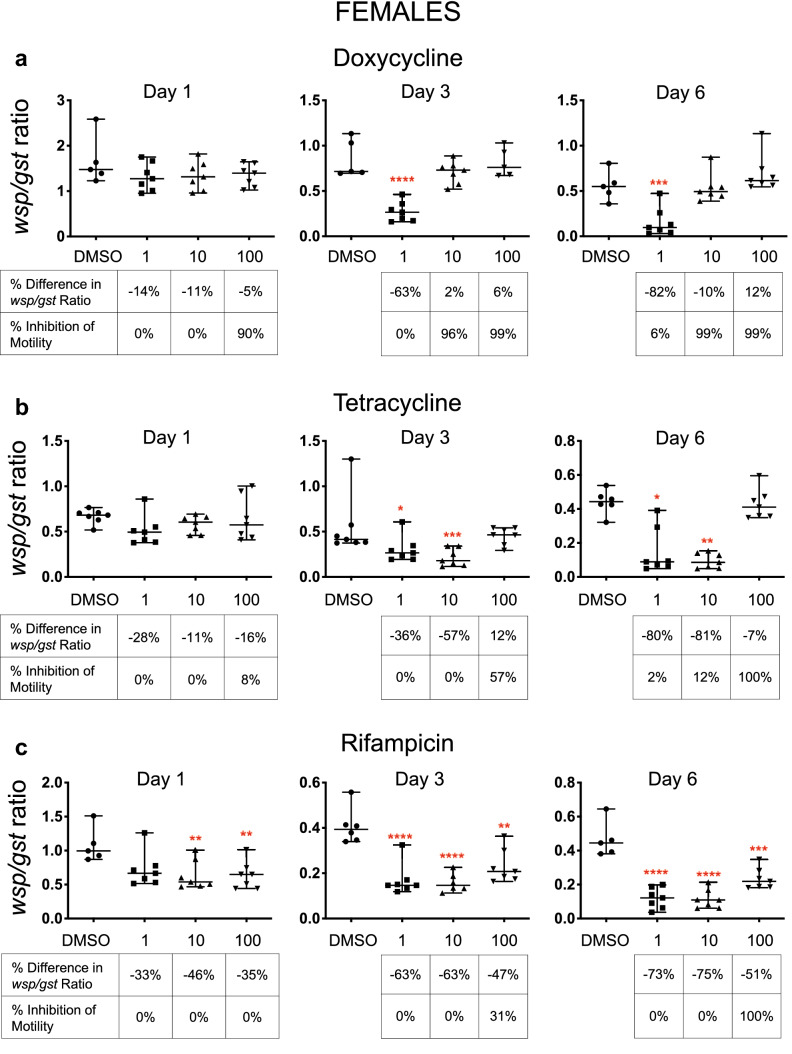
Time course experiment reveals antibiotics stop worm motility without a corresponding decrease in *Wolbachia* and *vice versa. *A time course experiment was conducted to determine the effects of doxycycline, tetracycline and rifampicin on *B. pahangi* females and *Wolbachia* titers at low (1 µM), intermediate (10 µM) and high (100 µM) concentrations at three time points (Day 1, 3 and 6). Doxycycline (**a**) and tetracycline (**b**) decreased worm motility but *wsp/gst* ratios did not fall in response to high antibiotic concentrations; at lower concentrations, worm motility was not impacted but *wsp/gst* ratios were reduced. *Wolbachia* titers were measured by *wsp/gst* ratios; medians with 95% confidence intervals are shown. X-axis labels show antibiotic concentration in µM. Percent differences in *wsp/gst* ratios compared to DMSO controls are shown below each antibiotic concentration. Negative percentages signify a decrease in *Wolbachia* titers, and positive percentages indicate that titers were higher than controls. Percent inhibition of motility is shown below each antibiotic concentration: 0% inhibition indicates that worms were as motile as controls, and 100% inhibition indicates that the worms were fully immotile. Red asterisks indicate statistical significance of the difference between *wsp/gst* ratios in treated worms and DMSO controls. ****P < 0.0001, ***P < 0.001, **P < 0.01, *P < 0.05. Statistical significances of inhibition of motility are shown in Table S2 and a graphic illustration is shown in Fig. 3

To further investigate the effects of antibiotics on female and male *B. pahangi,* both *Wolbachia* titers and worm motility at multiple antibiotic concentrations were assessed over time. These time course experiments showed that high concentrations of doxycycline and tetracycline did not reduce *Wolbachia* titers, though lower concentrations did; 100 µM doxycycline did not cause a significant decrease in *Wolbachia* titers in female worms at any time point compared to control worms, yet worm motility was inhibited by 90% on Day 1 and worms were moribund by Day 3 (99% inhibition of motility) (Fig. 2a; Additional file 1: Fig. S4, Table S2). At 10 µM, motility was inhibited by 96% on Day 3 and 99% on Day 6, also without significant reduction in *Wolbachia*. However, 1 µM doxycycline reduced *Wolbachia* titers by 63% on Day 3 and 82% on Day 6, although worms remained motile for the duration of the assay. Similar results were observed for male *B. pahangi* motility and for the *Wolbachia* titers at high and low concentrations of doxycycline (Additional file 1: Fig. S5 and S6, Table S2).

Tetracycline fully inhibited worm motility only when worms were exposed to 100 µM tetracycline for 6 days. At this high concentration, worms were immotile but *Wolbachia* titers were similar to those of control worms (Fig. 2b; Additional file 1: Fig. S7, Table S2). At the lower antibiotic concentrations (1 and 10 µM), *Wolbachia* titers were significantly reduced compared to those from control worms on Days 3 and 6, despite showing active motility. Thus, while both doxycycline and tetracycline showed the expected dose response relationship in terms of motility, the inverse relationship was found for *Wolbachia* titer.

### Rifampicin reduced worm motility only at high concentrations but *Wolbachia* titers were reduced at all concentrations in time course experiment

**Fig. 3:**
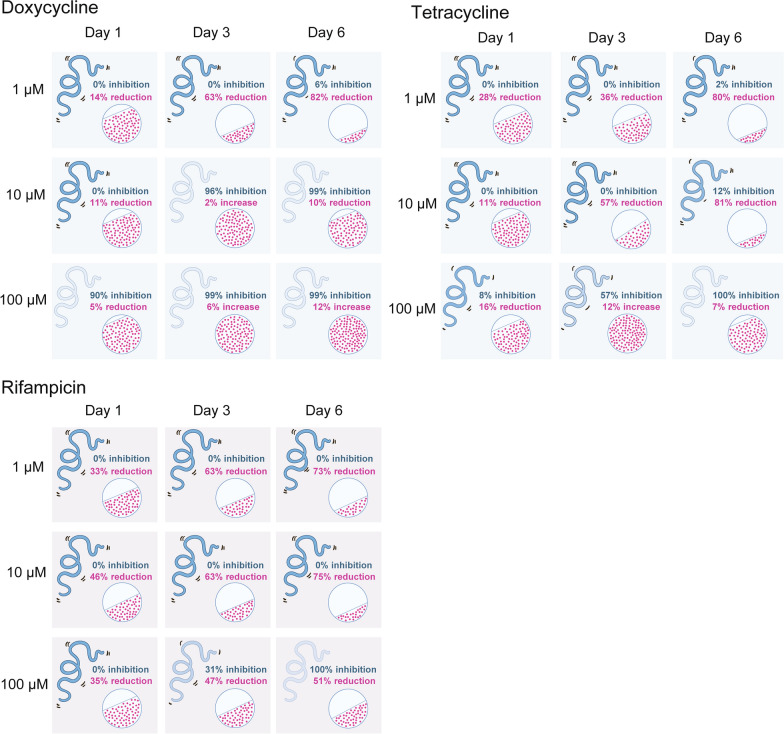
Illustration summarizing the Eagle effect on the endosymbiont *Wolbachia *in its worm host. The illustration depicts the worm and *Wolbachia* response to doxycycline, tetracycline and rifampicin. With doxycycline and tetracycline treatment worms become moribund at high concentration despite the high numbers of *Wolbachia*. Relative inhibition of worm motility is in blue (top), and relative changes in *Wolbachia* titers are in red (bottom). Worm motility is represented by the drawing of the worm: darker blue worms indicate more motility, and lighter blue worms indicate inhibited motility. *Wolbachia* titers are represented by red dots within the circle and are proportional to the *Wolbachia* titers normalized to controls

Rifampicin was also used in the time course experiment to assess the relationship between female and male worms and *Wolbachia* titers (Fig. 2c; Additional file 1: Fig. S5, S8 and S9, Table S2). Each concentration of rifampicin tested reduced *Wolbachia* titers but only the highest concentration inhibited motility. Worms treated with 100 µM rifampicin were moribund by Day 6. Rifampicin reduced *Wolbachia* titers at all concentrations starting as early as Day 1 for female worms and Day 3 for male worms. By Day 6 rifampicin reduced *Wolbachia* titers by 50% or more compared to control worms at all concentrations. Figure 3 summarizes the effects that doxycycline, tetracycline and rifampicin have on worms (motility) and *Wolbachia* numbers (% reduction) compared to control worms.

### Novel anti-*Wolbachia* compounds show trends similar to approved antibiotics

**Fig. 4:**
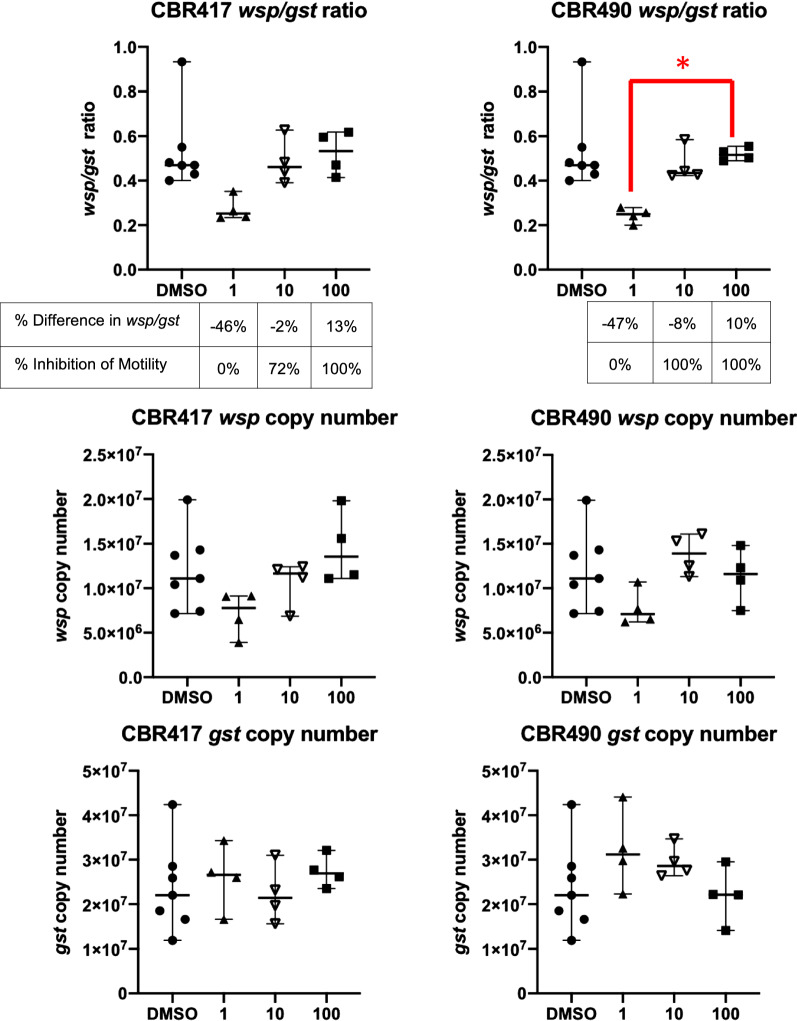
Quinazolines CBR417 and CBR490 are effective in killing worms at high concentrations and reduce *Wolbachia* numbers at low concentrations. *B. pahangi* adult females were treated with two novel anti-*Wolbachia* compounds, CBR417 and CBR490, for 3 days, *in vitro.* These compounds showed worm killing without *Wolbachia* reduction at 10 and 100 µM and worm survival with reduced *Wolbachia* titers at 1 µM. X-axis shows compound concentrations in µM. The same DMSO control worms were used as a comparator for both CBR417 and CBR490. Both compounds showed a positive correlation between antibiotic concentration and *wsp/gst* ratio (*r* ≥ 0.6) and a negative correlation between worm motility and *wsp/gst* ratio (*r* ≤ −0.9). The difference between 1 and 100 µM CBR490 was statistically significant (*P < 0.05). Inhibition of worm motility compared to DMSO controls at 10 and 100 µM for both compounds was statistically significant (*P* < 0.0001)

The novel quinazolines CBR417 and CBR490 were tested on *B. pahangi* female worms in a 3-day assay (Fig. 4). Both CBR417 and CBR490 induced the Eagle effect in *Wolbachia* titers, but as would be expected, inhibition of worm motility increased with compound concentration. Both compounds completely inhibited motility at 100 µM. Treatment with 10 µM CBR417 led to 72% inhibition of motility, while 10 µM CBR490 also inhibited motility by 100%. Similar to doxycycline and tetracycline, *Wolbachia* titers were not reduced even though worms were no longer motile at these concentrations. Conversely at the lowest concentration, 1 µM, *Wolbachia* titers were reduced by approximately 50% compared to the levels found in the controls but worms remained motile.

### Confocal microscopy confirms *Wolbachia *reduction in the distal tip cell when exposed to antibiotic treatment

**Fig. 5:**
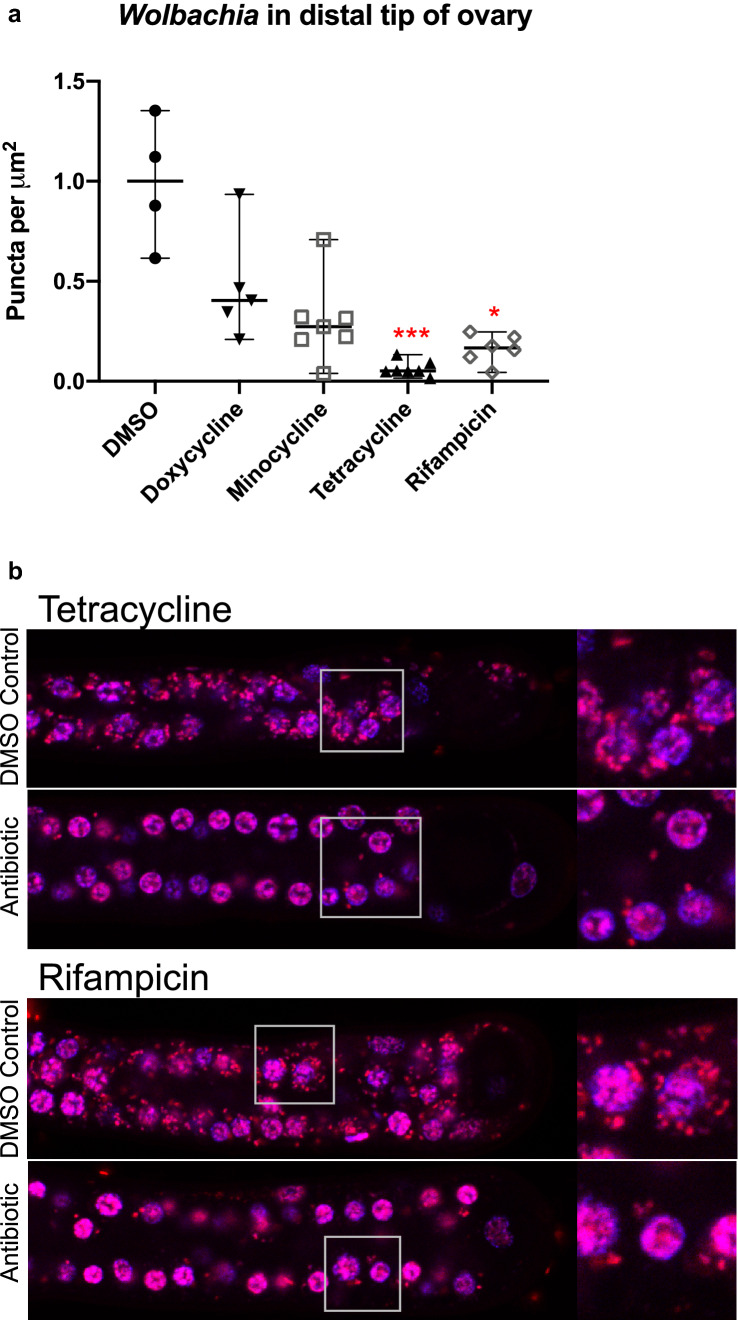
*Wolbachia* were depleted in the distal tip region of worm ovaries. Worms were treated with 10 µM doxycycline, minocycline, tetracycline and rifampicin for 6 days. Negative controls contained 1% DMSO in culture media. **a** Graph shows medians with 95% confidence intervals. ***P < 0.001; *P < 0.05. **b** Images of the distal tip region of *B. pahangi* ovaries from worms treated *in vitro* with tetracycline and rifampicin showing the elimination of *Wolbachia* in worm ovaries. Panels on the right are high magnification images of the boxed regions in the distal tip region. *Wolbachia* are the red puncta stained with propidium iodide and DAPI; the nuclei of host cells (worm cells) in the ovaries are stained blue/magenta by DAPI

Confocal microscopy of ovaries removed from worms treated with 10 µM doxycycline, minocycline, tetracycline and rifampicin revealed that there were lower numbers of *Wolbachia* in the distal tip region compared to those from worms in the control group. Figure 5 shows low and high magnification fluorescence images of fixed and stained untreated worms and worms treated with 10 µM tetracycline and rifampicin. Tetracycline significantly reduced the number of *Wolbachia* by 95% compared to the controls (*P* < 0.001); rifampicin also significantly reduced *Wolbachia* by 83% (*P* < 0.05). Although doxycycline and minocycline had lower *Wolbachia* titers (60 and 73%, respectively) compared to control worms, the reductions were not statistically significant.

## Discussion

While testing known antibiotics and novel anti-*Wolbachia* compounds with *Brugia pahangi* adult worms *in vitro*, we observed a surprising pattern: *Wolbachia* killing occurred at low antibiotic concentrations but *Wolbachia* survived when treated with higher concentrations. The IC_50_ and time course experiments showed that high concentrations of antibiotics failed to clear *Wolbachia* from the adult *Brugia pahangi* worms, while low concentrations decreased *Wolbachia* titers. This phenomenon, known as the Eagle effect, was first described by Eagle [40], who found that *Staphylococcus aureus*, *Enterococcus faecalis* and group B and C *Streptococcus* survived penicillin treatment at concentrations above an optimal point. Since this initial report, the Eagle effect has been reported in numerous species of bacteria and fungi treated with antibiotics across multiple classes [41–43, 56], but to our knowledge this is the first case in which the Eagle effect occurs with the endosymbiont, *Wolbachia*, in its worm host.

Although the underlying mechanisms that drive the Eagle effect are not known, investigators have suggested various possibilities to explain the increased survival of bacteria when treated with antibiotics at concentrations above the minimum inhibitory concentration (MIC), including antibiotic interference with bacterial autolytic enzymes, bacterial tolerance (bacteria transiently remain viable when exposed to high antibiotic concentrations) and the presence of non-replicating persister populations [42, 43]. In the *Wolbachia* endosymbiont/filarial worm relationship, it is possible that one or more of these mechanisms may be at play. Since *Wolbachia* are obligate intracellular bacteria, antibiotics must first pass through cells of the worm host to enter bacterial cells. It is possible that high concentrations of antibiotics such as doxycycline cause direct damage to host cells, which signal *Wolbachia* to initiate replication to maintain their population or to enter a protective, dormant “persister” state to reduce susceptibility to antibiotics. An analogous process occurs in adherent invasive *Escherichia* *coli* that are triggered to enter a persister state by the stressful conditions of the phagolysosome when phagocytosed by macrophages [57]. Lower antibiotic concentrations may be insufficient to cause damage to the worm cells, thus allowing the antibiotics to infiltrate the bacteria before signaling mechanisms can be engaged.

In an *in vivo* study by Gunderson et al. [39], *Wolbachia* titers were initially reduced following rifampicin treatment but then returned to normal levels 8 months later. They reported that populations of *Wolbachia* found within clusters were not reduced by antibiotic treatment, but that *Wolbachia* in the areas surrounding the clusters were eliminated, suggesting that these clusters contained *Wolbachia* in a protected state. It is possible that the clusters are affording protection for the *Wolbachia* and act as a privileged site in the worm that allows the bacteria to persist and contribute to the Eagle effect.

Given that worm motility was inhibited at high concentrations independently of *Wolbachia* killing, the antibiotics’ effect on worms was likely due to off-target effects. For instance, the tetracycline class of antibiotics (doxycycline, tetracycline, and minocycline) achieve their bacteriostatic effects by binding to the 30S ribosomal subunit, thereby inhibiting bacterial protein synthesis [58, 59], but they are also known to have effects on eukaryotic cells, e.g. inhibit mitochondrial function in both *Wolbachia*-infected and -uninfected *Drosophila simulans* [60], influence apoptosis [61, 62] and inhibit matrix metalloproteinases [63]. *Brugia* are known to have metalloproteinases that play important physiological roles, and the inhibition of these enzymes may play a role in worm killing [64]. These off-target drug effects may also affect worm survival when rifampicin is given at high concentrations *in vitro*. Rifampicin is known to induce reactive oxygen species (ROS) in bacteria [65, 66] in addition to inhibiting bacterial RNA polymerase. The mechanism of action is not yet known for the new quinazolines, CBR417 and CBR490, but these compounds resulted in findings similar to those of rifampicin *in vivo*. Animal studies have shown that these compounds decreased *Wolbachia* titers by 90–99% compared to vehicle controls [33, 36, 39], which suggests that worms recovered from treated animals may correspond to those worms that were exposed to low (1–10 µM) concentrations of antibiotics in the present *in vitro* study. Thus, worms recovered *in vivo* receive what may be the equivalent of low doses *in vitro*. However, further pharmacokinetic studies are needed to evaluate how *in vitro* results relate to *in vivo* studies.

## Conclusions

Observation of the Eagle effect in *Wolbachia* suggests a common underlying mechanism that allows for diverse bacterial and fungal species to persist despite exposure to high concentrations of antimicrobial compounds. Further investigation into the Eagle effect in the *Wolbachia*-*Brugia* endosymbiotic relationship may shed light on conserved mechanisms by which bacteria evade antibiotic treatment and lead to improved treatments for both filarial and bacterial infections.

## Supplementary Information

**Additional file 1: Figure S1.** IC_50_s of adult female and male worm motility on Day 6 of *in vitro* assays. **Figure S2.** Female IC_50_s *wsp* and *gst* copy numbers. **Figure S3.** Male IC_50_s *wsp* and *gst* copy numbers. **Figure S4.** Female doxycycline time course *wsp* and *gst* copy numbers. **Figure S5.** Male time course assay results. **Figure S6.** Male doxycycline time course *wsp* and *gst* copy numbers. **Figure S7.** Female tetracycline time course *wsp* and *gst* copy numbers. **Figure S8.** Female rifampicin time course *wsp* and *gst* copy numbers. **Figure S9.** Male rifampicin time course *wsp* and *gst* copy numbers. **Table** **S1.** Statistical significance of changes in *wsp/gst* ratios in IC_50_ assays. **Table** **S2.** Statistical significance of changes in *wsp/gst* ratios in time course assays.

## Data Availability

All data generated and analyzed during this study are included in this published article and its supplementary information files.

## Abbreviations

MDA: Mass drug administration
mf: Microfilariae
MTT: Thiazolyl blue tetrazolium bromide
