# Supplementary material for: The Eagle effect in the Wolbachia-worm symbiosis
Source: Parasit Vectors. 2021 Feb 24;14:118. doi: 10.1186/s13071-020-04545-w (PMC7905570; doi:10.1186/s13071-020-04545-w)
Supplement: Supplementary file 1 — Additional file 1: Figure S1. IC50s of adult female and male worm motility on Day 6 of in vitro assays. Figure S2. Female IC50s wsp and gst copy numbers. Figure S3. Male IC50s wsp and gst copy numbers. Figure S4. Female doxycycline time course wsp and gst copy numbers. Figure S5. Male time course assay results. Figure S6. Male doxycycline time course wsp and gst copy numbers. Figure S7. Female tetracycline time course wsp and gst copy numbers. Figure S8. Female rifampicin time course wsp and gst copy numbers. Figure S9. Male rifampicin time course wsp and gst copy numbers. Table S1. Statistical significance of changes in wsp/gst ratios in IC50 assays. Table S2. Statistical significance of changes in wsp/gst ratios in time course assays. [file 13071_2020_4545_MOESM1_ESM.pdf]

Figure S1: IC_{50}s of adult female and male worm motility on Day 6 of *in vitro* assays

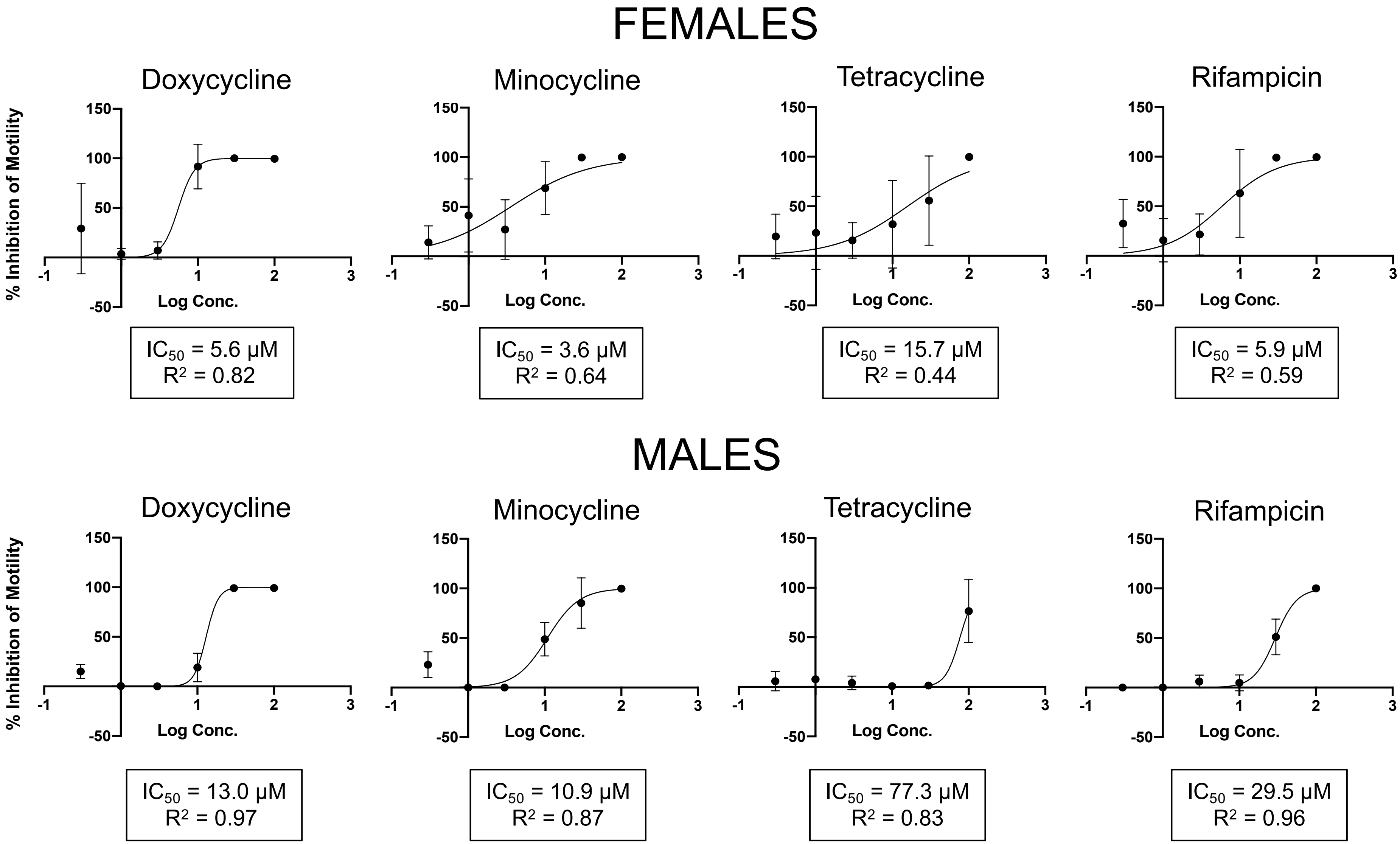

Fig. S1: Worm motility showed the expected relationship between antibiotic concentration and motility; i.e. higher antibiotic concentrations were associated with lower worm motility and lower antibiotic concentrations were associated with higher worm motility. Error bars show standard deviation of the mean.

Figure S2: Female IC_{50}s *wsp* and *gst* copy numbers

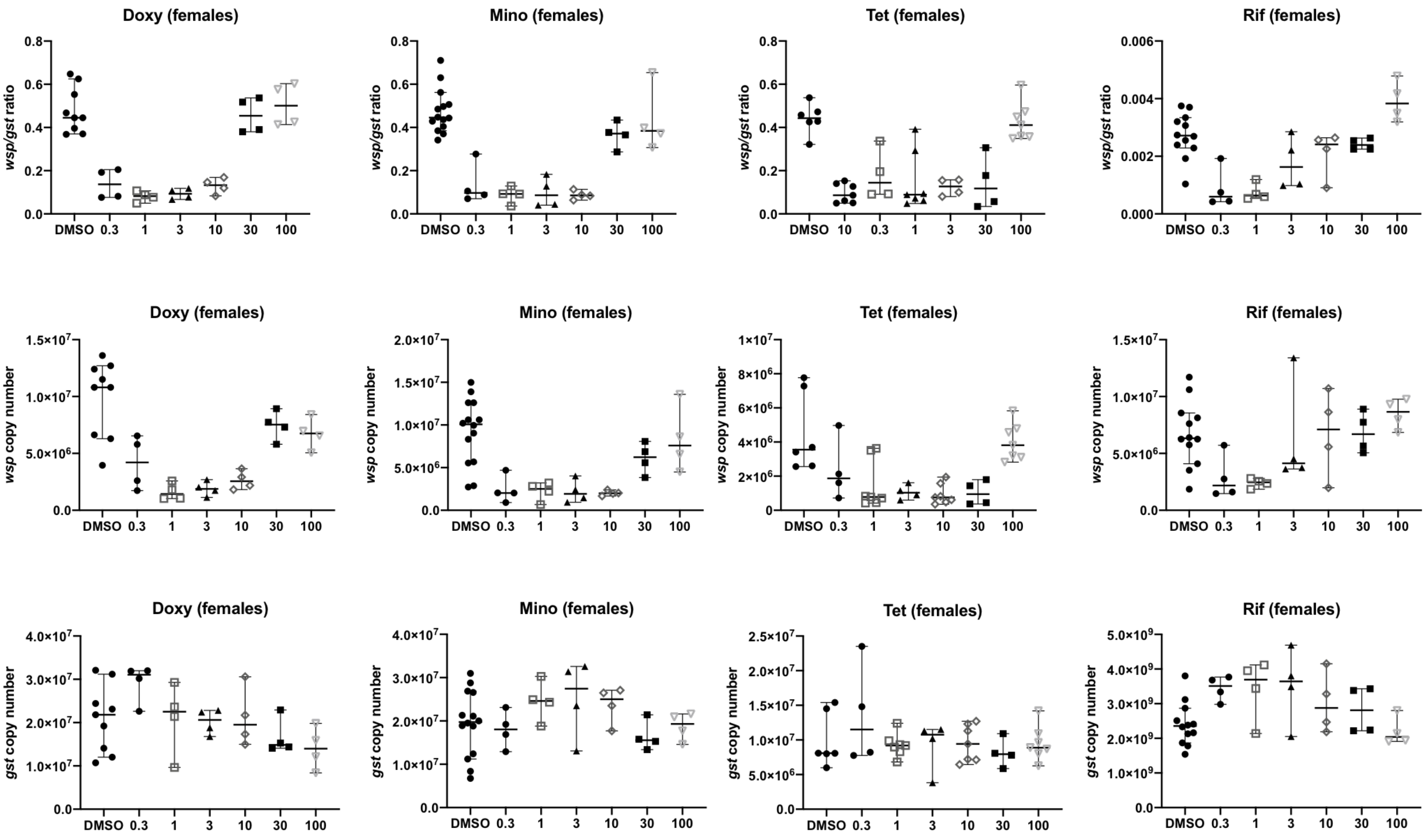

Figure S3: Male IC_{50}s *wsp* and *gst* copy numbers

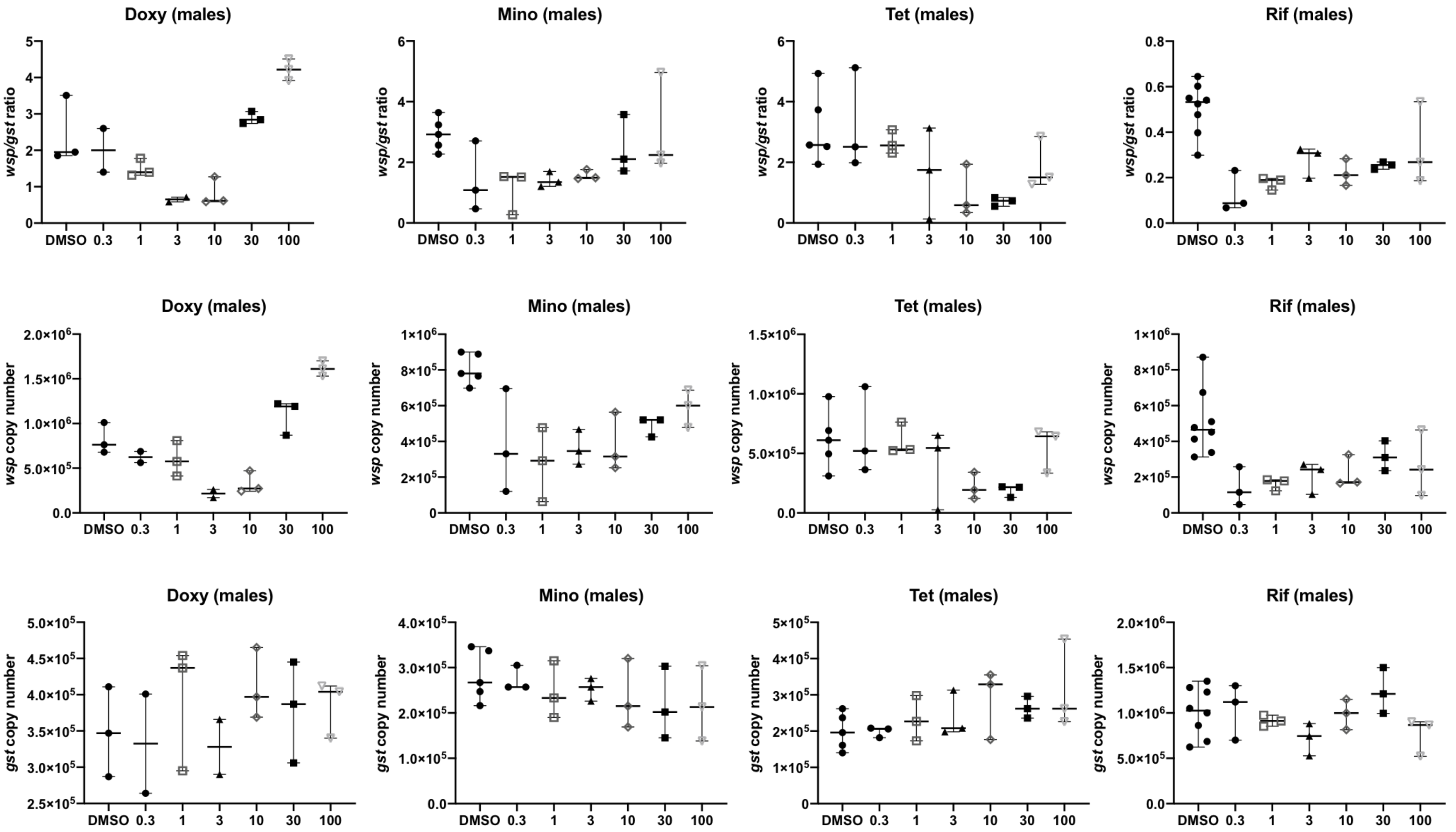

Figure S4: Female doxycycline time course *wsp* and *gst* copy numbers

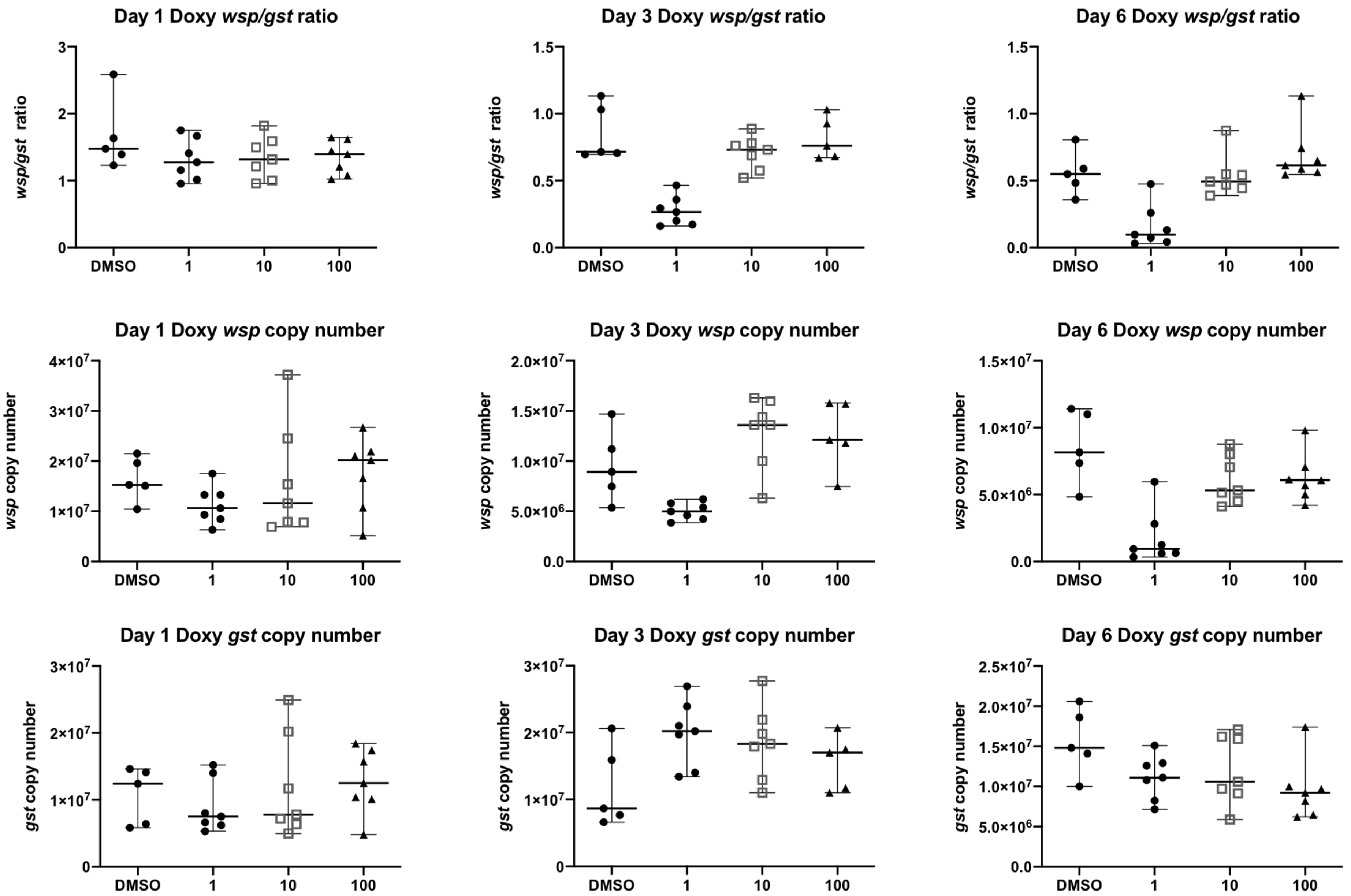

Figure S5: Male time course assay results

MALES

Doxycycline

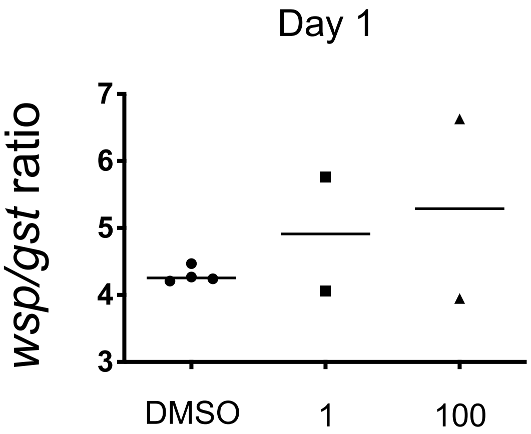

	1	100
% Difference in *wsp/gst* Ratio	15%	24%
% Inhibition of Motility	6%	44%

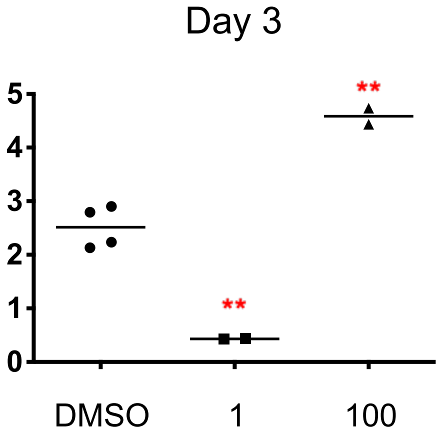

	1	100
	-83%	82%
	0%	99%

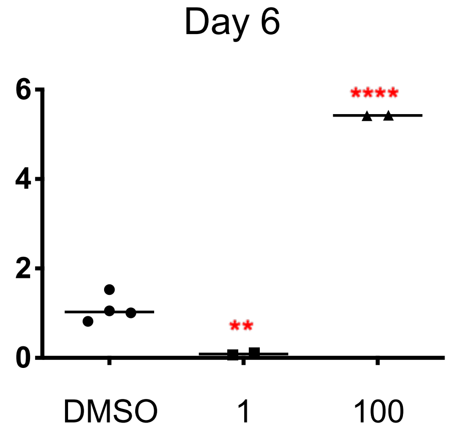

	1	100
	-92%	427%
	0%	100%

Rifampicin

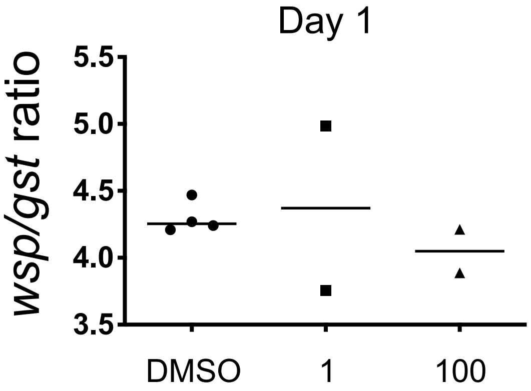

	1	100
% Difference in *wsp/gst* Ratio	3%	-5%
% Inhibition of Motility	11%	18%

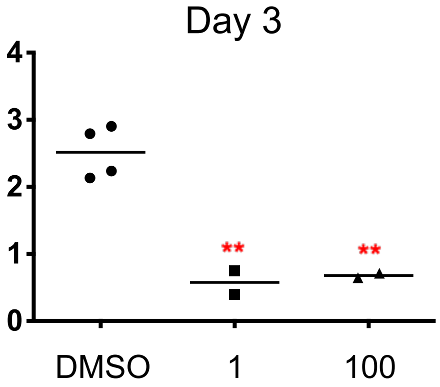

	1	100
	-77%	-73%
	3%	19%

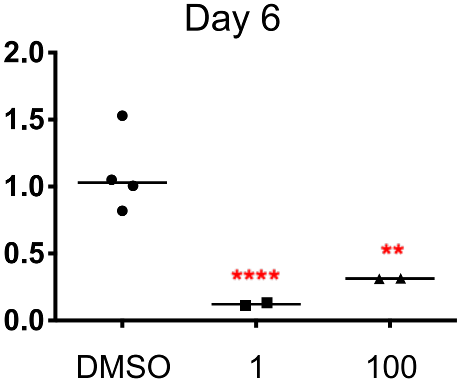

	1	100
	-88%	-69%
	0%	100%

Fig. S5: Male time course assay results

A time course experiment was conducted to determine the effects of doxycycline, tetracycline and rifampicin on *B. pahangi* adult males and *Wolbachia* titers at low (1 μM) and high (100 μM) concentrations at three time points (Day 1, 3 and 6). *Wolbachia* titer and motility of male worms treated with doxycycline and rifampicin followed a similar pattern as female worms. Low concentration doxycycline reduced *Wolbachia* titers but did not affect worm motility. High concentration doxycycline killed worms, but *Wolbachia* titers increased 4X by Day 6. Rifampicin reduced *Wolbachia* titers on Day 3 and Day 6 at both low and high concentrations, but worms were only killed with 100 μM rifampicin on Day 6. *Wolbachia* titers were measured by *wsp/gst* ratio; medians are shown. X-axis labels show antibiotic concentration in μM. The percentage difference in *wsp/gst* ratio as compared to DMSO controls is shown below each antibiotic concentration. Negative percentages signify a decrease in *Wolbachia* titers and positive percentages indicate that titers were higher than controls. The percent inhibition of motility is shown below each antibiotic concentration as well; 0% inhibition indicates that worms were as motile as controls and 100% inhibition indicates that the worms were fully immotile. Red asterisks indicate statistical significance of the difference between *wsp/gst* ratios in treated worms and DMSO controls. ****P < 0.0001, ***P < 0.001, **P < 0.01, *P < 0.05. Statistical significance of inhibition of motility can be found in Supplemental Table S2.

Figure S6: Male doxycycline time course *wsp* and *gst* copy numbers

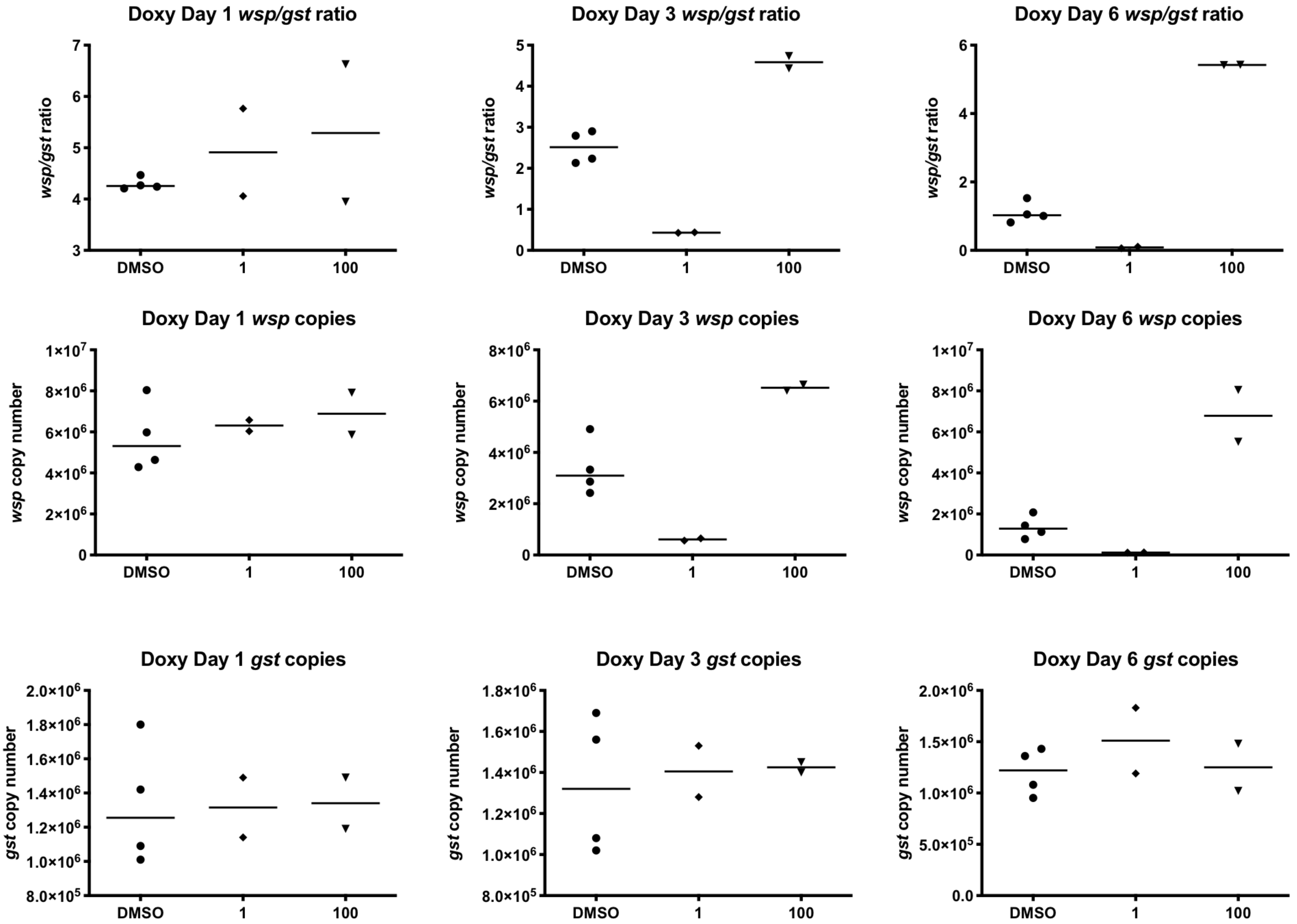

Figure S7: Female tetracycline time course *wsp* and *gst* copy numbers

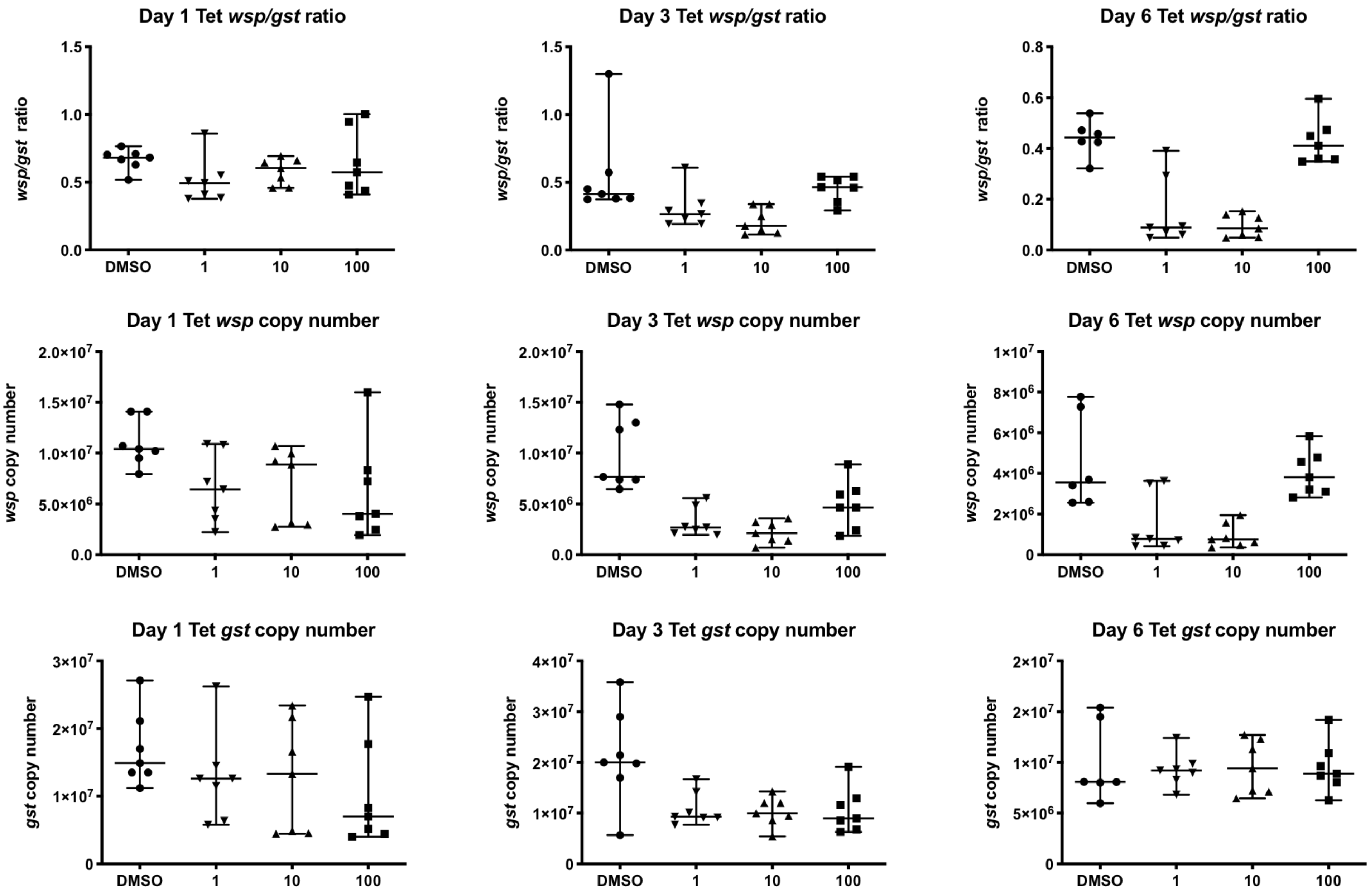

Figure S8: Female rifampicin time course *wsp* and *gst* copy numbers

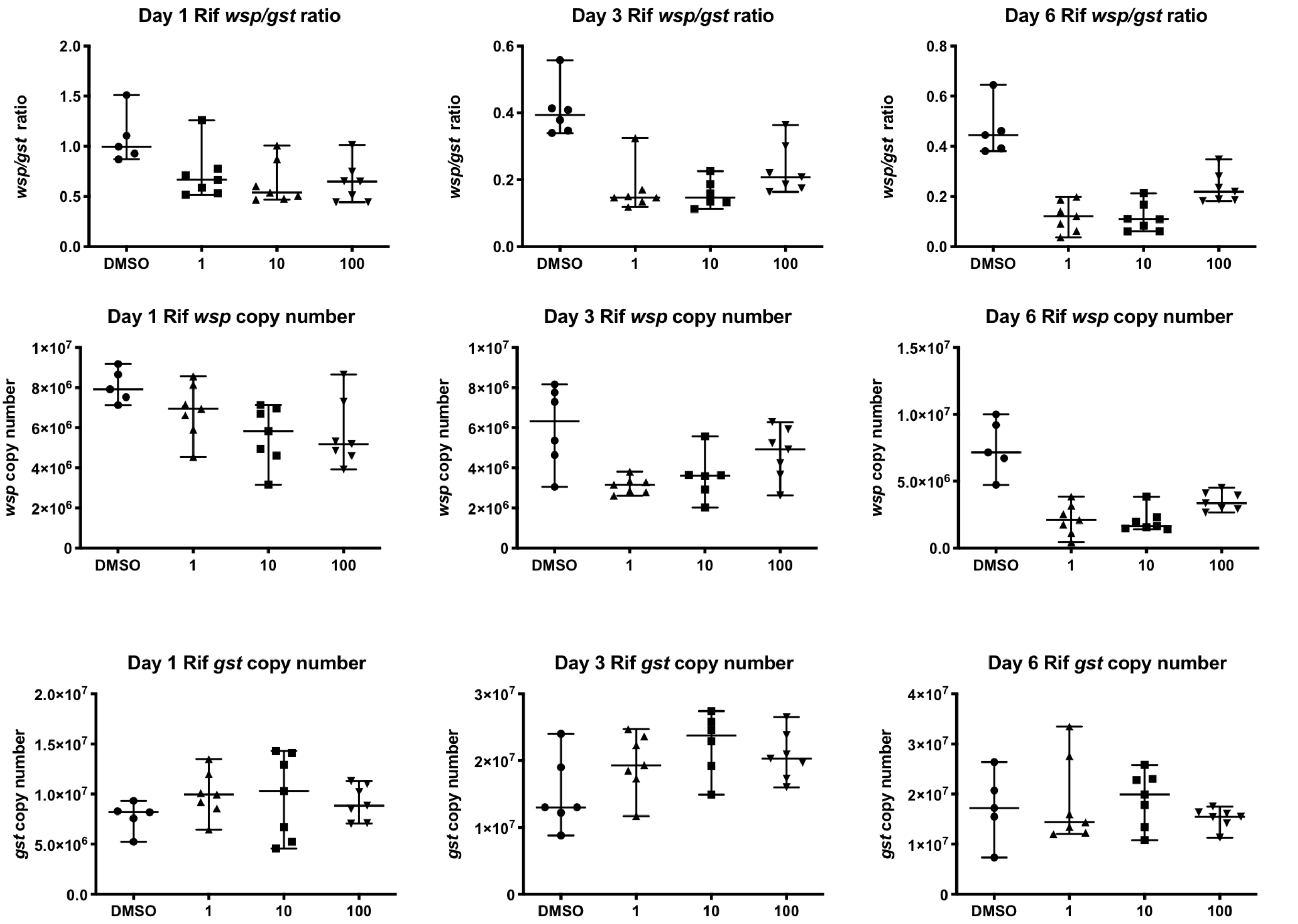

Figure S9: Male rifampicin time course *wsp* and *gst* copy numbers

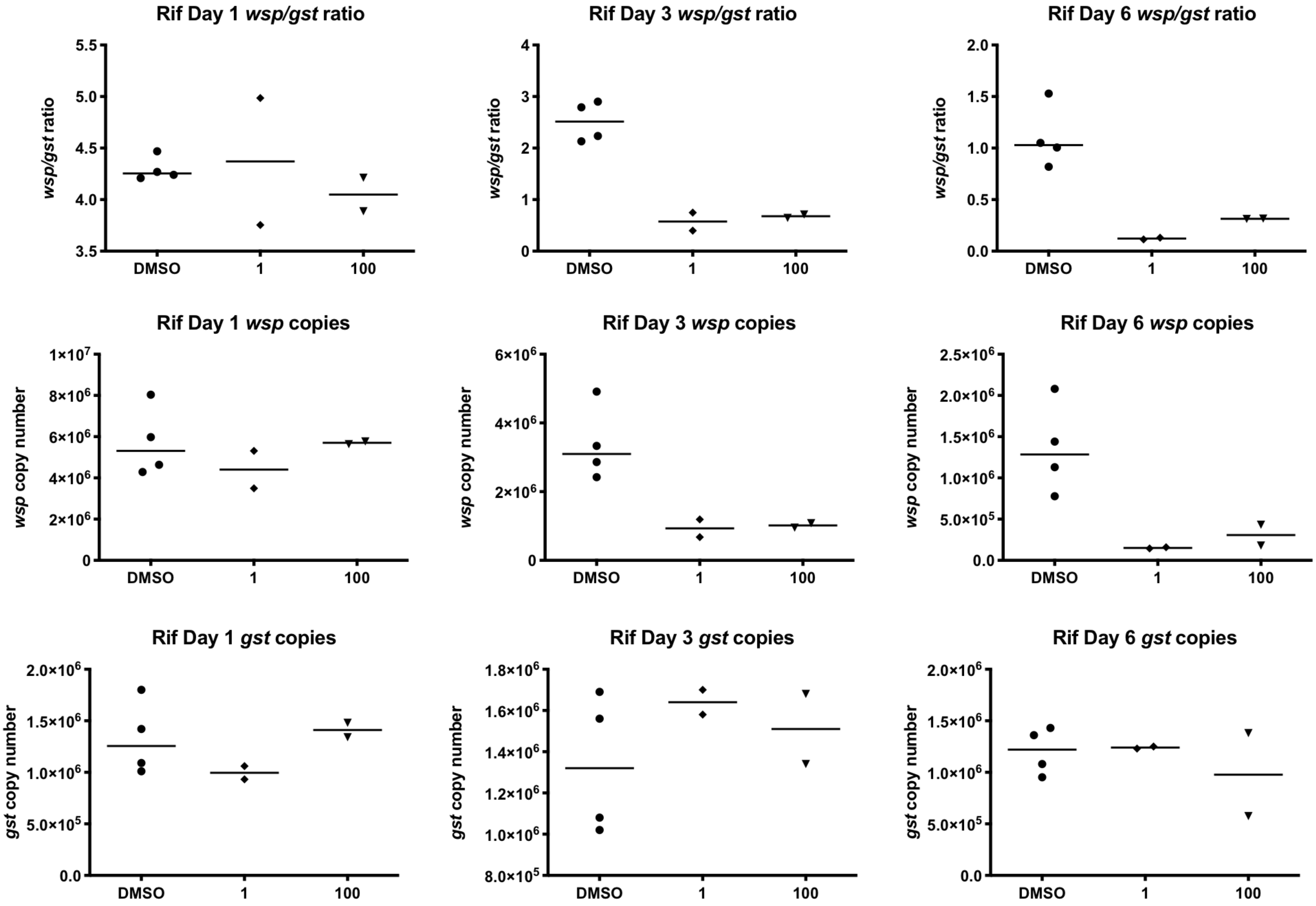

SUPPLEMENTARY TABLE S1

FEMALES

Conc (µM)	Doxycycline % diff in *wsp/gst*	Doxycycline Significance	Doxycycline Motility IC_{50}	Minocycline % diff in *wsp/gst*	Minocycline Significance	Minocycline Motility IC_{50}	Tetracycline % diff in *wsp/gst*	Tetracycline Significance	Tetracycline Motility IC_{50}	Rifampicin % diff in *wsp/gst*	Rifampicin Significance	Rifampicin Motility IC_{50}
**100**	13%	ns		-14%	ns		-7%	ns		41%	ns	
**30**	2%	ns		-17%	ns		-74%	*	15.7 µM	-12%	ns	
**10**	-70%	ns	5.6 µM	-81%	**	3.6 µM	-81%	**		-11%	ns	5.9 µM
**3**	-79%	*		-81%	**		-71%	ns		-40%	ns	
**1**	-81%	**		-79%	**		-80%	*		-76%	*	
**0.3**	-69%	ns		-78%	**		-67%	ns		-78%	*	

MALES

Conc (µM)	Doxycycline % diff in *wsp/gst*	Doxycycline Significance	Doxycycline Motility IC_{50}	Minocycline % diff in *wsp/gst*	Minocycline Significance	Minocycline Motility IC_{50}	Tetracycline % diff in *wsp/gst*	Tetracycline Significance	Tetracycline Motility IC_{50}	Rifampicin % diff in *wsp/gst*	Rifampicin Significance	Rifampicin Motility IC_{50}
**100**	116%	ns		-23%	ns		-42%	ns	77.3 µM	-50%	ns	
**30**	46%	ns	13.0 µM	-28%	ns	10.9 µM	-72%	ns		-52%	ns	29.5 µM
**10**	-69%	ns		-49%	ns		-77%	ns		-60%	ns	
**3**	-67%	ns		-54%	ns		-32%	ns		-42%	ns	
**1**	-28%	ns		-48%	ns		-1%	ns		-64%	**	
**0.3**	3%	ns		-63%	ns		-2%	ns		-83%	**	

Table S1: Statistical significance of changes in *wsp/gst* ratios in IC_{50} assays

Table S1 shows the percent difference in *wsp/gst* ratios between antibiotic treated worms and DMSO control worms, along with their statistical significance. Negative percentage differences indicate a decrease in *Wolbachia* titers; positive percentages indicate higher *Wolbachia* titers than controls. ns = not significant ($P > 0.05$), * $P < 0.05$; ** $P < 0.01$. Red lines demarcate the concentrations between which the IC_{50} falls.

SUPPLEMENTARY TABLE S2

FEMALES

	Doxycycline [1 µM]			Doxycycline [10 µM]			Doxycycline [100 µM]		
	Day 1	Day 3	Day 6	Day 1	Day 3	Day 6	Day 1	Day 3	Day 6
% difference in *wsp/gst* ratios	-14%	-63%	-82%	-11%	2%	-10%	-5%	6%	12%
Significance	ns	p < 0.0001	p < 0.001	ns	ns	ns	ns	ns	ns
% difference in motility	0%	0%	-6%	0%	-96%	-99%	-90%	-99%	-99%
Significance	ns	ns	ns	ns	p < 0.0001	p < 0.0001	p < 0.0001	p < 0.0001	p < 0.0001

	Tetracycline [1 µM]			Tetracycline [10 µM]			Tetracycline [100 µM]		
	Day 1	Day 3	Day 6	Day 1	Day 3	Day 6	Day 1	Day 3	Day 6
% difference in *wsp/gst* ratios	-28%	-36%	-80%	-11%	-57%	-81%	-16%	12%	-7%
Significance	ns	p < 0.05	p < 0.05	ns	p < 0.001	p < 0.01	ns	ns	ns
% difference in motility	0%	0%	-2%	0%	0%	-12%	-8%	-57%	-100%
Significance	ns	ns	ns	ns	ns	ns	ns	p < 0.0001	p < 0.0001

	Rifampicin [1 µM]			Rifampicin [10 µM]			Rifampicin [100 µM]		
	Day 1	Day 3	Day 6	Day 1	Day 3	Day 6	Day 1	Day 3	Day 6
% difference in *wsp/gst* ratios	-33%	-63%	-73%	-46%	-63%	-75%	-35%	-47%	-51%
Significance	ns	p < 0.0001	p < 0.0001	p < 0.01	p < 0.0001	p < 0.0001	p < 0.01	p < 0.01	p < 0.001
% difference in motility	0%	0%	0%	0%	0%	0%	0%	-31%	-100%
Significance	ns	ns	ns	ns	ns	ns	ns	p < 0.0001	p < 0.0001

MALES

	Doxycycline [1 µM]			Doxycycline [100 µM]		
	Day 1	Day 3	Day 6	Day 1	Day 3	Day 6
% difference in *wsp/gst* ratios	15%	-83%	-92%	24%	82%	427%
Significance	ns	p < 0.01	p < 0.01	ns	p < 0.01	p < 0.0001
% difference in motility	-6%	0%	0%	-44%	-99%	-100%
Significance	ns	ns	ns	p < 0.0001	p < 0.0001	p < 0.0001

	Rifampicin [1 µM]			Rifampicin [100 µM]		
	Day 1	Day 3	Day 6	Day 1	Day 3	Day 6
% difference in *wsp/gst* ratios	3%	-77%	-88%	-5%	-73%	-69%
Significance	ns	p < 0.01	p = 0.0001	ns	p < 0.01	p < 0.01
% difference in motility	-11%	-3%	0%	-18%	-19%	-100%
Significance	ns	ns	ns	p < 0.01	p < 0.05	p < 0.0001

Table S2: Statistical significance of changes in *wsp/gst* ratios in time course assays

Table S2 shows the percent difference in *wsp/gst* ratios between antibiotic treated worms and DMSO control worms and the percent decrease in motility, along with the statistical significance. Statistically significant differences are colored green.
